# Phycospheric Native Bacteria *Pelagibaca bermudensis* and *Stappia* sp. Ameliorate Biomass Productivity of *Tetraselmis striata* (KCTC1432BP) in Co-cultivation System through Mutualistic Interaction

**DOI:** 10.3389/fpls.2017.00289

**Published:** 2017-03-06

**Authors:** Jungsoo Park, Bum Soo Park, Pengbin Wang, Shailesh K. Patidar, Jin Ho Kim, Sae-Hee Kim, Myung-Soo Han

**Affiliations:** Department of Life Science, College of Natural Sciences and Research Institute for Natural Sciences, Hanyang UniversitySeoul, South Korea

**Keywords:** *Tetraselmis*, phosphate, phycosphere, growth promoting bacteria, lipid, biomass, biofuel

## Abstract

Effective sustainable algal cultivation techniques are essential for mass production of the marine microalga *Tetraselmis* for biofuel and array of co-products. The phycospheric communities affect the microalgal growth and metabolism through various allelochemical and nutrient interactions; hence, their potential to affect the quantity and quality of both biomass and bioproducts is significant. In the present study, we have screened the phycospheric communities of biofuel producing *Tetraselmis striata* (KCTC1432BP). A total of 26 bacterial strains were isolated and identified from the phycosphere of *T. striata* mass culture. Then, each bacterial strain was tested in co-cultivation conditions with *T. striata* for evaluating its growth promoting and inhibitory effects. Among these all strains, two promising strains (*Pelagibaca bermudensis* KCTC 13073BP and *Stappia* sp. KCTC 13072BP) were selected because of their maximum growth promoting effects and mutualistic interactions. The growth rate, biomass productivity, lipid contents, and fatty acids were analyzed during their combined growth in O3 media and compared with axenic growth of *T. striata*. Later, growth promoting mechanisms in the co-cultivation environment were investigated for these promising bacterial strains under replete and limited conditions of nutrients (nitrate, phosphate, and vitamin B_12_). The growth promoting potential of *P. bermudensis* was illustrated by the two fold enhancement in biomass productivity. These bacteria are promising for microalgal cultivation without any negative effects on the native seawater bacterial communities, as revealed by next generation sequencing analysis. This study represents, to date, the first report highlighting the role of phycospheric growth promoting bacteria of promising biofuel feedstock *T. striata*.

## Introduction

Microalgae have drawn extensive global attention for their possible application as third generation biodiesel production since some of them are rich in saturated and mono-unsaturated fatty acids (Green et al., 2015; Mitra et al., 2015; Patidar et al., 2016; Wang et al., 2016). In addition to generating huge biomass through low commercial nutrient inputs, it is essential to integrate feasible biofuel production with many allied products. However, biofuels from microalgae are not commercially viable yet, and up to what extent the potential of biofuels from microalgae could be realized in the near future is still a matter of contention (Do Nascimento et al., 2013). Several obstacles to commercialization need to be overcome by key research breakthroughs, including strain selection, lipid content improvement, cultivation system design and microalgal culture harvesting (Wang et al., 2016).

Marine microalga *Tetraselmis striata* KCTC12432BP was selected as a candidate in this study because it is well-known to have high lipid content and fast growth (Chisti, 2007; Teo et al., 2014). In addition, *T. striata* used in this study has been exploited for large scale mass culture in South Korea due to their resistance to a wide range of temperatures (Cho et al., 2014). Also, *T. striata* has been widely used as a source of nutrition for invertebrates in aquaculture for decades (Peña and Villegas, 2005). Thus, suitable engineering is essential to enable the economical biomass production of *T. striata* significant for cultivation of *T. striata*. The growth of microalgae is affected and influenced by the culture conditions such as light intensity, nutrient limitation, temperature, pH, CO_2_ concentration and salinity (Patidar et al., 2014; Mitra et al., 2015). To date, significant number of studies have been reported on the optimum conditions needed for attainment of higher growth of *T. striata* (Moheimani, 2013; Khatoon et al., 2014). Among them, an often-overlooked factor that could play a critical role in the whole process of microalgal biofuel production is the bacterial communities associated with the biofuel-producing microalgae. Previously, the studies have revealed that the bacteria contribute substantially to the growth of phytoplankton (Kodama et al., 2006), highlighted by the algal–bacterial symbiosis (Geng and Belas, 2010; Seyedsayamdost et al., 2011). In other words, bacteria could provide a broader array of substances, e.g., specific compounds such as nutrients and vitamins like growth-promoting substances in addition to the respiratory product such as CO_2_ (Kazamia et al., 2012; Do Nascimento et al., 2013). Thus, such added bacteria may increase microalgal growth rates, and enhance metabolic yields through the synthesis of growth-promoting compounds (Croft et al., 2005; Park et al., 2008). There have been reports of role of bacteria in enhancing microalgal production. As an example, *Rhizobium* sp., is a part of a community of bacteria which promote plant growth and collectively termed as plant growth promoting rhizobacteria (PGPR) (Kloepper et al., 1980; Lee et al., 2013). Recent studies on PGPR revealed that they significantly enhance microalgal growth (Hernandez et al., 2009; Lee et al., 2013). According to reports mentioned above, it is likely that the inclusion of symbiotic bacterial treatment in future mass cultivation systems may lead to cost-reduction.

On the other hand, there are only few studies related to growth enhancement of algae by bacteria and, most studies suggest a highly species specific interaction in algal cultivation systems (Le Chevanton et al., 2013; Kim et al., 2014; Wang et al., 2016). A search for specific algal growth promoting bacterial group with microalgae is needed because axenic algal cultures only exist under strict laboratory conditions and even in relatively non-sterile environment (Andersen, 2005; Bolch et al., 2011; Ramanan et al., 2015). The engineering of phycosphere with the desired bacteria would lead to biomass product avenues with higher yield through the beneficial biochemical interactions.

Thus, the main objective of this paper is to (1) find mutualistic bacteria from phycosphere, to (2) unveil the plausible role of algal–bacterial growth promoting effects, to (3) produce higher biomass with desired fatty acids in culture system, and ultimately, to (4) to check the growth behavior of growth promoting bacteria together with *T. striata* in xenic seawater conditions.

## Materials and Methods

### Preparation of Culture of *T. striata* and Bacteria

#### Axenic Culture of *T. striata*

*Tetraselmis striata* (KCTC12432BP) used in this study was obtained from the Department of Biological Engineering, Inha University, Incheon, South Korea. Initially, to eliminate bacteria associated with *T. striata* and to obtain axenic cultures, at least six generations of single colony were isolated and purified on the sterile F/2 (Guillard, 1975) media plate (final concentration: 50 mg/L streptomycin plus 50 mg/L ampicillin in autoclaved agar medium). The purified colonies after antibiotic treatment were inoculated in O3 media (McIntosh and Cattolico, 1978) and, stored at 20°C exposed to cool-white fluorescent lamps (50 μmol photons m^-2^ s^-1^) on a 12 h:12 h light : dark photoperiod. Prior to the inoculation of microalgae in each experiment, the culture was checked for any contamination of bacteria using DAPI staining and observing it under fluorescence microscope (BX51, Olympus, Japan). Axenic condition of *T. striata* cultures was also confirmed by using fluorescence microscopy during the experiments (Supplementary Figure S1).

#### Isolation of Bacteria from *Tetraselmis* Culture

The total of 26 bacterial isolates listed in **Table 1** were obtained from *Tetraselmis* mass culture as the following protocol: the original water sample (0.5 mL) was spread on the Marine Broth (MB, Difco) media plate and incubated at 25°C. Thereafter, phenotypically different colonies obtained from the plates were transferred to the fresh MB plates, followed by purification of bacterial isolates.

**Table 1:** Preliminary screening test for isolated 26 bacterial strains obtained from the phycosphere of *Tetraselmis striata* for their growth promoting and algicidal effects on various microalgae (the “-” shows algicidal and “+” shows growth promoting effects.

Bacteria strain	Growth promoting effects				
	
	*T. striata*	*Heterosigma*	*Chattonella*	*Amphidinium* sp.	*Dunaliella*
	KCTC12432BP	*akashiwo*	*marina*		*tertiolecta*
HYYH-1409-2	-0.33	0.21	-0.07	-0.04	-0.43
HYYH-1409-3	-0.07	0.11	-0.80	0.37	-0.03
HYYH-1409-4-1	-0.19	0.34	-0.30	0.04	-0.64
**HYYH-1409-4-3**	**1.66**	**0.21**	-**0.60**	-**0.04**	-**0.67**
HYYH-1409-7	-0.27	0.74	-0.83	0.26	-0.16
HYYH-1409-8	-0.38	0.32	-0.87	-0.11	-0.01
HYYH-1409-10-1	-0.48	-0.98	-0.97	-0.83	0.10
HYYH-1409-11-1	-0.42	0.42	-0.90	-0.41	-0.07
HYYH-1409-11-2	-0.33	1.63	-0.90	-0.02	-0.27
HYYH-1409-12	-0.46	0.34	-0.87	0.02	-0.28
HYYH-1409-13	-0.19	0.05	-0.60	-0.04	-0.46
HYYH-1409-16-1	0.00	0.74	-0.83	0.26	-0.16
HYYH-1409-16-2	2.29	0.37	-0.90	-0.37	-0.73
HYYH-1409-17-1	2.48	1.11	-0.63	-0.07	-0.76
**HYYH-1409-17-3**	**3.04**	**1.53**	-**0.83**	**0.30**	-**0.43**
HYYH-1410-18-1	-0.04	1.21	-0.73	0.23	-0.58
HYYH-1410-18-2	0.00	-0.05	-0.97	-0.17	-0.40
HYYH-1410-18-3	0.16	0.26	-0.73	0.04	-0.25
HYYH-1410-19	-0.19	0.68	-0.83	0.04	-0.09
HYYH-1410-20	-0.20	-0.26	-0.97	0.00	-0.10
HYYH-1410-21	-0.18	-0.05	-0.97	-0.17	-0.40
HYYH-1410-23	-0.15	-0.21	-0.93	0.00	-0.27
HYYH-1410-25	-0.19	0.53	-0.70	0.52	-0.55
HYYH-1410-26	-0.07	0.37	-0.93	-0.28	-0.76
HYYH-1410-28	0.76	0.26	-0.97	0.26	-0.21
HYYH-1410-39	1.43	-1.00	-1.00	-1.00	-1.00


*Bacteria with growth effects of ≥1.5 and ≤-0.4 are regarded as growth-promoting and algicidal bacteria, respectively). Bold indicates Strains selected as *Tetraselmis* growth-promoting bacteria through the screening test.*

#### Co-cultivation of Isolated Bacteria with Microalgae

Bacteria were cultured in Marine broth (Difco, South Royal, USA) media at 30°C with 150 rpm shaking for 72 h, harvested through centrifugation (3700 × *g* at 4°C for 10 min) and washed three times with fresh O3 medium. The bacteria were enumerated using DAPI staining followed by fluorescence microscopy prior to inoculation in O3 media. The initial density of ∼1 × 10^7^ cells mL^-1^ of bacteria was adjusted under standard sterile conditions in each of the experimental flasks. Microalgal cells (5 × 10^5^ mL^-1^) and the bacterial suspensions were inoculated at the same time in O3 culture media. All the cell counts were only considered from duplicate, triplicate or quadruplicate subsamples (*n* = 4, 6, 8), respectively.

### Screening and Testing for Growth-Promoting Bacteria

Preliminary screening tests were performed to select TGPB which could enhance the growth of *Tetraselmis* among the 26 isolated bacterial colonies. The bacteria isolated from phycosphere of *T. striata* were also investigated for their algicidal or growth promoting effects on other microalgae (*Heterosigma akashiwo*, *Chattonella marina*, *Amphidinium* sp., *Dunaliella tertiolecta*) listed in **Table 1**. Growth-promoting bacteria were screened by comparing the cell density of axenic *T. striata* in duplicate controls and treatments (on inoculation of isolated bacteria) by cultivating them for 10 days. The growth effect was measured by following Eq. (1):

$$\text{Growth effect=}({A^{\prime }}_{\left(\text{T-Treatment}\right)}-{\text{A}}_{\left(\text{T-Control}\right)})/{\text{A}}_{\left(\text{T-Control}\right)}$$

where A_(*T-Control*)_ (Cells mL^-1^) and A′_(*T-Treatment*)_ (Cells mL^-1^) are the cell abundance of *Tetraselmis* in the control and treatment, respectively, and T is the inoculation time (day). Bacteria with growth effects of ≥1.5 and ≤-0.4 are regarded as growth-promoting and algicidal bacteria, respectively (Park et al., 2016).

### Identification of *Tetraselmis* Growth Promoting Bacteria (TGPB)

For the two selected strains, *Pelagibaca bermudensis* KCTC 13073BP and *Stappia* sp. KCTC 13072BP, DNA was extracted with DNeasy Plant Mini Kit (QIAGEN, South Korea). In turn, 16S rDNA of two bacteria were PCR amplified using the bacterial universal primers 27F/1525R. The amplification was carried out on a thermocycler (T100, Thermal Cycler, BIO-RAD) according to the following procedure: 5 min at 95°C, then 38 cycles including 30 s at 95°C, 30 s at 56°C and 1 min at 72°C, and a final step of 10 min at 72°C. PCR products were checked on a 1.5% agarose electrophoresis gel. The amplified lengths of DNA were then sequenced at ‘BIONICS’ (South Korea^1^). Sequence alignments were done by BioEdit software and phylogenetic analyses were performed on the basis of neighbour-joining (NJ) methods using MEGA version 6.

### Determination of Biomass Productivity, Dry Weight, Lipid Contents, and Enumeration of Cell Numbers

Biomass productivity of both *Tetraselmis* and co-cultivated *Tetraselmis* with *P. bermudensis* was determined by DCW measurement. The independent experiment with the same experimental conditions repeated for the measurement of biomass productivity wherein each experimental set kept separately for harvesting on different growth interval. An amount of 100 mL of each experimental culture flask was used for harvesting. Culture harvesting was conducted by using centrifugation (3700 × *g* for 10 min), and wet biomass kept for drying at 70°C in a drying oven for 24 h. Total DCW of each sample was measured by subtracting weight of vacant tube from the final weight of dry cell containing tube. Biomass productivity (mg L^-1^ day^-1^) is calculated by the Eq. (2) below.

$$\text{Biomass productivity}(\text{mg}{\text{L}}^{-1}{\text{day}}^{-1})=\text{DCW}(\text{mg/L})/\text{t}\left(\text{day}\right)$$

DCW is dry cell weight, and *t* is the culture time in days. Total lipids were extracted from known weight of dried biomass by using a solvent mixture of chloroform, methanol (2:1 by volume) according to the Folch method (Folch et al., 1957) and lipid productivity was calculated by using the Eq. (3) and Eq. (4) given below.

Total lipid(%)=(Lipid weight(mg/L)/DCW)×100⁢

$$\text{Lipid productivity}(\text{mg}{\text{L}}^{-1}{\text{day}}^{-1})=\text{Biomass productivity}\times \text{Total lipid}(\%).\,$$

Cell counting was simultaneously conducted by the following protocol: subsamples for *Tetraselmis* were fixed in Lugol’s solution (final concentration 1%), and then growth of green algae was determined by enumerating the cells observed using hemocytometer under a light microscope at 200x magnification (BX51, Olympus, Japan). The growth was monitored by counting the cells using hemocytometer after suitable dilution in triplicate subsamples.

### Fatty Acid Composition

The fatty acid composition was determined using the protocol supplied by MIDI Inc. through the GC-FID. The operational conditions are described by Tran et al. (2009). Each fatty acid was identified and quantified based on comparing the retention times and peak areas (Tran et al., 2009).

### Bacterial Abundance

To determine the cell density of the bacteria, subsamples were fixed in 1% glutaraldehyde (final concentration), filtered onto black 0.2 μm. Isopore membrane filters (Millipore, Germany), and stained with 4′,6-diamidino-2-phenylindole (DAPI). DAPI stained cells were counted at 1000x magnification using fluorescence microscopy (BX51, Olympus, Japan).

### Growth of TGPB in O3 Media with and without *T. striata*

The growth of each selected individual species *P. bermudensis* and *Stappia* sp. investigated in O3 media without inoculation of *T. striata* and, growth of co-culture (*Tetraselmis* and *Stappia* sp.) were compared. The cell abundance on each successive day of the growth observed and experimental sets were compared. The abundance of *T. striata* and bacterial density was measured using the microscope (BX51, Olympus, Japan) according to the protocol described in Section “Determination of Biomass Productivity, Dry Weight, Lipid Contents, and Enumeration of Cell Numbers” and “Bacterial Abundance,” respectively.

### Changes of Bacterial Composition in Co-cultivation System in Xenic Seawater (*Ex situ* Introduction)

Seawater obtained from Incheon (west coast of South Korea) was filtered onto 2 μm. Isopore membrane filters (Millipore, Germany) to eliminate some cysts and eggs of marine animals except for bacterial community. Then, bacterial density of filtered seawater was counted by DAPI. O3 media grown *T. striata* was transferred to filtered (2 μm) seawater. Thereafter, *P. bermudensis* and *Stappia* sp. in different ratio (initial percentage of TGPB against bacteria in nature, e.g., 10, 50, 90%) were inoculated into the xenic seawater containing *T. striata* independently. In addition to algal growth rates, DNA from all subsamples (duplicates) was extracted. Subsequently, the changes of bacterial compositions via next generation sequencing (NGS) technology were analyzed by following the protocol of ‘MACROGEN’ (MiSeq, South Korea^2^).

### Nutrient Acquisition between *Tetraselmis* and TGPB in Co-cultivation System

#### *T. striata* under Nutritional Stress (Nitrogen, Phosphate, and Vitamin B_12_ Limitation) and Optimum Nutrient Conditions

To investigate whether TGPB (*P. bermudensis* and *Stappia* sp.) extracellular substances have any significant role on the *Tetraselmis* culture (O3 medium), the respective nutrient limited medium (nitrate, phosphate, and vitamin B_12_) was used for comparison. After inoculation of TGPB into nutrient limited media, algal growth was determined by cell counting.

#### Specific Phosphate Release in Absence of Nutrient Uptake by Live *T. striata* Cells (Experiment with Metabolically Inactive *T. striata*)

*Tetraselmis striata* was frozen with liquid nitrogen when cells could reach stationary phase for investigating further nutrient interactions under phosphate-limited condition. Metabolically inactive cells of *T. striata* disabled to uptake any phosphate were used; thereby consumable phosphate released by TGPB could be detected. No growth of metabolically inactive algal cells was observed by culturing in fresh O3 medium up to 20th day. Thus, the metabolically inactive cells were utilized in the co-culture experiment to investigate interaction between *T. striata* and TGPB under phosphate-limited conditions and compared with positive controls.

#### Determination of Phosphate in Co-cultivation Culture Medium during the Growth

To analyze the changes in phosphate concentration after the addition of TGPB, TP, inorganic phosphate, and organic phosphate were measured using the ascorbic acid reduction followed by phospho-molybdenic spectrophotometric method (Grasshoff et al., 2009). The samples for the dissolved organic and inorganic phosphate analysis obtained by filtering these from 0.22 μm Whatman filter paper. The TP (total particulate + non particulate phosphate) was measured without filtering the samples containing cells of bacteria and/or microalgae (after inoculation) on different growth interval. The samples for TP were digested and pretreated as per the method mentioned by Grasshoff et al. (2009) prior to analysis.

## Results and Discussion

### Screening Test for Selection of *Tetraselmis* Growth Promoting Bacteria (TGPB)

The growth promoting effects of the promising bacteria on *T. striata* were compared to axenic *Tetraselmis* culture. Four bacterial isolates namely HYHH-1409-4-3, HYHH-1409-16-2, HYHH-1409-17-1, and HYHH-1409-17-3 out of the total 26 bacterial isolates were observed to enhance the growth of *T. striata* (**Table 1**). Based on the preliminary screening test, two bacteria were selected and phylogenetically identified as *P. bermudensis* (HYHH-1409-4-3) and *Stappia* sp. (HYHH-1409-17-3) (Supplementary Figure S2). Bacterial treatment (*P. bermudensis* and *Stappia* sp.) enhanced algal growth up to 1.7 times and three times over control (axenic), respectively. Although, two other bacterial isolates (HYHH-1409-16-2 and HYHH-1409-17-1) also showed growth promoting effect (**Table 1**) on *T. striata*, they were excluded for next level of experimentation due to same phylogenetical position as *Stappia* sp. (HYHH-1409-17-3).

Two isolated bacteria (*P. bermudensis* and *Stappia* sp.) from *Tetraselmis* mass culture have been designated as TGPB and may have mutualistic relationship with *T. striata*. These two bacteria belong to the Alphaproteobacteria class which was found to be dominantly distributed in phycosphere (Sapp et al., 2007) and also considered as having plant growth promoting function, i.e., providing inorganic nutrients such as nitrate and phosphorus (Cavalca et al., 2010; Karagöz et al., 2012). To the best of our knowledge, *P. bermudensis* and *Stappia* sp. have been investigated and reported for the first time for their growth promoting effects on *T. striata*.

### Algal Growth, Biomass Productivity, and Lipid Productivity of *T. striata* on Using of TGPB

**FIGURE 1:**
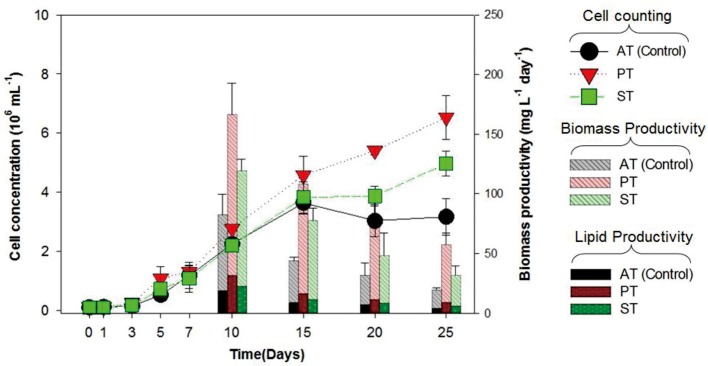
**Cell concentration, biomass productivity (mg L^-1^ day^-1^) and lipid productivity (mg L^-1^ day^-1^) of *Tetraselmis striata* with co-cultivation of TGPB (AT = Axenic *T. striata*, PT = Mixed culture of *Pelagibaca bermudensis* and *T. striata*, ST = Mixed culture of *Stappia* and *T. striata*)**.

The growth of *T. striata* (**Figure 1**) was measured through monitoring cell concentration after inoculating TGPB and also lipid content, in addition to dry weight were evaluated on successive day of growth. The results showed that *P. bermudensis* stimulated up to two-fold increase in cell density (6.5 × 10^6^ mL^-1^) of *T. striata* on the 25th day compared to the control (axenic *T. striata*) while reaching the stationary phase. However, biomass productivity was the maximum up to 166 mg L^-1^ day^-1^ at 10th day. *Stappia* sp. also increased cell density of *T. striata* up to 4.9 × 10^6^ mL^-1^ at 25th day and maximum biomass productivity (119 mg L^-1^ day^-1^) was at 10th day, compared to cell density of axenic *Tetraselmis* reached (3.1 × 10^6^ mL^-1^) at 25th day. The maximal biomass productivity (83 mg L^-1^ day^-1^) was found at 10th day in *Stappia* sp. treated culture. Although the highest algal cells number (6.5 × 10^6^ mL^-1^) observed at 25th day after inoculation of *P. bermudensis* in co-cultivation system, upon considering the maximum biomass productivity, it is suggested that the best harvesting time should be from 10th to 15th day. According to **Figure 1**, the stationary phase of *T. striata* lasted longer in the bacterial treated groups, until the end of the culture periods (25 days). This showed that the survival rate of number of cells in late growth phase (15–25 days) was not restricted for a longer time due to stress mitigation through the uptake of nutrients released by the TGPB (Do Nascimento et al., 2013). The analyses of lipid content in the treated and axenic cultures suggested that the axenic cultures had better capacity to accumulate lipids. The percentage of lipid on cell dry weight basis in the bacterial treated groups (18 ± 3%) was relatively lower than control group (23 ± 2.3%) at 10th day, however, total biomass and lipid productivity were still substantially promoted by the bacterial treatment (**Figure 1**). The results of fatty acid composition revealed lesser variations. The results of fatty acid methyl ester (FAME) profiles exhibited approximately 35% palmitic acid (C16:0), 20% linolenic acid (C18:3), and 10% stearic acid (C18:0) as the dominant fatty acids along with abundance of other four different fatty acids (Supplementary Figure S3). Palmitic acid and stearic acid are known as common fatty acids for biodiesel production and these fatty acids can reinforce the quality of biodiesel (Mitra et al., 2015; Patidar et al., 2015, 2016). Since concentration of significant fatty acids was not compromised in the bacterial treatment group compared to control group, augmentation of lipid productivity could be ensured for transforming it to biodiesel production. Most of the species of *Tetraselmis* genus are well-known for biodiesel production (Van Thang Duong and Hall, 2015).

### Feasibility of Using TGPB in Xenic Seawater Conditions

**FIGURE 2:**
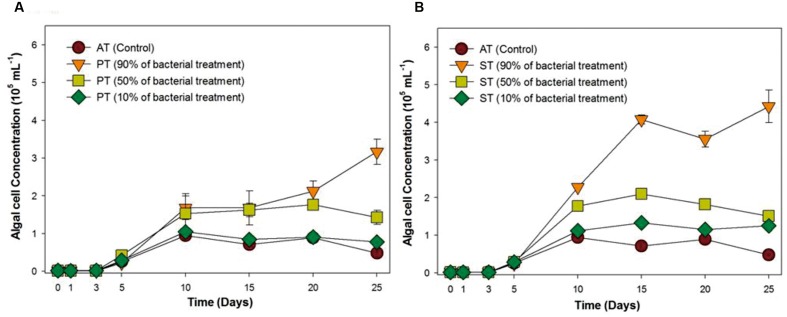
**Growth rates of *T. striata* (KCTC12432BP) in xenic seawater collected from Youngheng, South Korea.** The percentage (90, 50, and 10%) indicates initial inoculating density of **(A)** *P. bermudensis* and **(B)** *Stappia* species against native bacterial diversity in nature (AT = Axenic *T. striata*, PT = Mixed culture of *P. bermudensis* and *T. striata*, ST = Mixed culture of *Stappia* and *T. striata*).

Since the bacterial growth promoting effect on *T. striata* has proven under selective co-cultivation conditions in this study, it was necessary to prove whether these bacterial effects are applicable to field cultivation system, being exposed to a myriad of different native bacteria of seawater. Accordingly, serial inoculation of TGPB was adjusted (10, 50, and 90%) against natural cumulative bacterial density (10^6^ mL^-1^) (estimated through DAPI counting). In the results, interestingly, although 10% of initial concentration of TGPB (10^5^ ml^-1^) did now show notable algal growth effect, more than 50% of initial concentration of TGPB (10^6^ mL^-1^) inoculated group remarkably showed maximum growth of *T. striata* (**Figure 2**). This result indicated that the effectiveness of TGPB for *T. striata* in xenic seawater was even higher than the artificial O3 media. This is also suggested that the cumulative bacterial assemblage with dominant 50% of *P. bermudensis* inoculation on initial day in co-cultivation conditions could have stimulatory beneficial interaction within bacterial communities which may release some metabolites and nutrients providing net beneficiary effect to the microalgae. Nutrient exchange has been considered the most common type of algal bacterial interaction (Kouzuma and Watanabe, 2015). This knowledge led to the next experimental design under various nutrient limited conditions (nitrate, phosphate, vitamin B_12_) in the current study.

**FIGURE 3:**
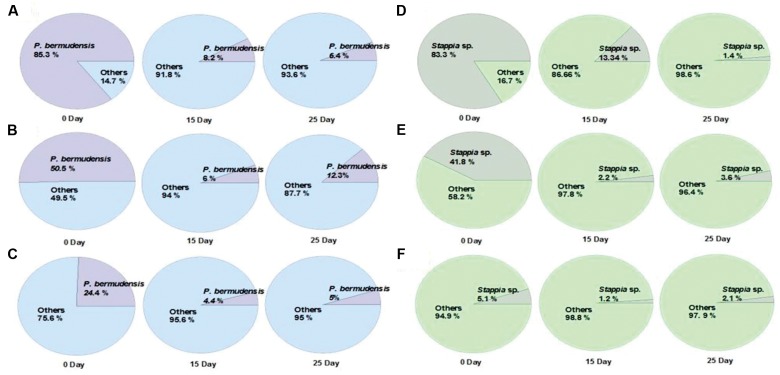
**Changes of the occupancy ratio of TGPB in xenic seawater (0.2 μm filtered) co-cultivated with *T. striata* during the growth** (**A** = 85.3% of *P. bermudensis* initial inoculation; **B** = 50.5% of *P. bermudensis* initial inoculation; **C** = 24.4% of *P. bermudensis* initial inoculation; **D** = 83.3% of *Stappia* sp. initial inoculation; **E** = 41.8% of *Stappia* initial inoculation; **F** = 5.1% of *Stappia* sp. initial inoculation).

The fate of TGPB inoculation while it competes with other native bacteria in xenic seawater was monitored by measuring the changes in occupancy ratio of each TGPB strain (**Figure 3**). However, the performance of these bacteria in terms of their growth is weaker in xenic environment compared to the designed co-cultivated conditions in O3 media. According to the results of NGS data, the percentage of TGPB decreased up to optimum occupancy ratio of 5∼12.3% (*P. bermudensis*) and 1.4∼3.6% (*Stappia* sp.) in total gene pool, regardless of their initial cell density (**Figure 3**). This implicated that the native bacterial assemblage slow down the growth of *ex situ* introduction of the TGPB but could not inhibit the net stimulatory effect on the *T. striata* as revealed by NGS data. Both two TGPB were not found in field seawater taken as control. However, the possibility of other growth promoting bacteria which could interact with *T. striata* for enhancement of growth cannot be ruled out since xenic seawater contains higher bacterial diversity than the bacteria screened from phycosphere.

Taken together, the higher initial concentration of TGPB (90 and 50%) promoted growth of *T. striata* dramatically in seawater (**Figure 2**), even though the initial density of TGPB was higher, it had reached up to the non-dominant concentration during successive experimental period by competing against other native bacteria in nature. Initial bacterial interactions to microalgae within phycosphere during the growth may include the strategy to create favorable environment by supplying a large amount of growth-promoting substances such as inorganic nutrients, vitamins, trace elements, chelators, and phytohormones (Wang et al., 2016). To reduce competing stress with other undesired bacteria, a stable combination of beneficial bacteria with TGPB is suggested to bolster its sustainability in future applications. Assemblage of TGPB would also prevent pathogenic and undesired exotic bacteria that invade mass microalgal culture (Cho et al., 2015). These results imply the possibility of exploiting bacteria for achieving higher biomass productivity in field water conditions. However, further study should be supported in a mesocosm-scale to ascertain factual economic benefits in the process of cultivation system at the field itself.

### Phosphate Released by TGPB Interacting Microalgae

**FIGURE 4:**
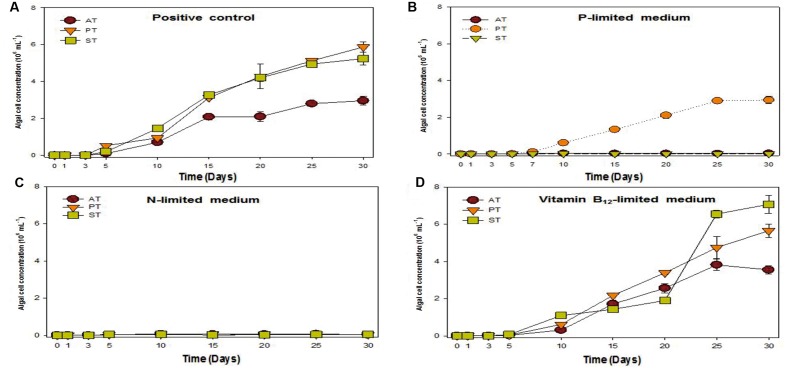
**Growth rates of *T. striata* (KCTC12432BP) in varied cultivation system under** **(A)** enriched nutrients conditions, **(B)** phosphate-limited conditions, **(C)** nitrate-limited conditions, and **(D)** vitamin B_12_-limited conditions, respectively (AT = Axenic *T. striata*, PT = Mixed culture of *P. bermudensis* and *T. striata*, ST = Mixed culture of *Stappia* and *T. striata*).

Previous research studies reported that symbiotic bacteria contribute to algal growth by supplying nutrients (Philippot et al., 2013; Kouzuma and Watanabe, 2015; Ramanan et al., 2016). *T. striata* with TGPB was co-cultured under phosphate, nitrate, and vitamin B_12_ limited condition (**Figure 4**) to unveil the nature of interaction. The results exhibited that the growth of *T. striata* co-cultured with TGPB was highly restrained under nitrate-limited condition. This indicated that nitrogen limitation hampers the growth of both *T. striata* as well as TGPB and both TGPB (*P. bermudensis* and *Stappia* sp.) could not supply organic nitrogen/inorganic nitrate to restore or complement the growth of *T. striata*. However, vitamin B_12_-lmited conditions reflected that response of the growth (cell concentration) in the phase of 0–20 days were significantly different from 20 to 30 days. Notably, *Stappia* sp. could enhance cell concentration of *T. striata* on 20–25 days possibly due to alternative substitute of vitamin supplementation in the medium or it was due to vitamin B_12_ supplementation on the same growth interval. Interestingly, the cell concentration of *Stappia* sp. treated *T. striata* in vitamin B_12_ was higher than the axenic *T. striata*. It was even higher (up to ∼7 × 10^6^ cells mL^-1^) than the *P. bermudensis* treated cultutre (up to ∼6 × 10^6^ cells mL^-1^) which could not show any remarkable beneficial effect in vitamin B_12_-limited condition (**Figure 4**).

Upon experimentation under phosphate-limited conditions, *P. bermudensis* could show growth promoting effects by enhancing cell concentration of *T. striata* in co-culture system. These results clearly indicated that *T. striata* treated with *P. bermudensis* grew up to 3 × 10^6^ mL^-1^, whereas growth of axenic *T. striata* was completely restricted under phosphate-limited condition (**Figure 4**). Hence, study was conducted in co-cultivation system to measure TP, inorganic phosphate, and organic phosphate to know the chemical speciation of phosphate sources. Dissolved inorganic phosphate in the medium of live *T. striata* culture as well as in the co-cultivation with TGPB remained undetected (Data not shown). Accordingly, metabolically inactive algal cells (*T. striata*) were prepared to rule out the possibility of phosphate-consumption by live microalgae cells.

To understand the phenomenon, the different phosphate forms in the metabolically inactive algal cultures were measured under P-limited conditions. The result exhibited that DIP was remarkably increased (up to 12.76 μM mL^-1^) from 0 to 20 days during the experiment (**Table 2**) in *P. bermudensis* added culture, while no increment of DIP was found in axenic metabolically inactive *Tetraselmis* culture. The inorganic phosphate concentration was not detected in culture of metabolically inactive axenic *T. striata* up to 15 days of the growth, but, it was detected in minimal concentration (0.13 μMol) at 20th day. The DIP concentration in *P. bermudensis* treated culture of metabolically inactive *T. striata* was 5.13, 6.12, and 12.76 μMol at 5th, 10th, 15th day, respectively. The DOP concentration in *P. bermudensis* treated culture of metabolically inactive *T. striata* was 3.31, 0.81, and 1.42 μMol at 5th, 10th, 15th day, respectively. These results indicate that phosphate were mobilized and produced by *P. bermudensis* for the stress mitigation and, withstand themselves for the growth. In the light of the results, *P. bermudensis* could supply phosphate to *T. striata* under P-limited condition. Therefore, *P. bermudensis* was established as a source of phosphate under co-cultivation condition.

**Table 2:** Phosphate concentration (μM) in the metabolically inactive *Tetraselmis striata* containing cultures inoculated together with growth promoting bacteria in phosphate-limited O3 media (all phosphate measurement on 0 day was carried out after inoculation of cells).

	Axenic *T. striata* (Control)			*P. bermudensis* – *T. striata*			*Stappia* sp. – *T. striata*		
			
	TP	DIP	DOP	TP	DIP	DOP	TP	DIP	DOP
0 Day	0.57 ± 0.01	ND	ND	22.91 ± 0.01	**ND**	**ND**	9.09 ± 0.1	ND	ND
5 Day	0.50 ± 0.02	ND	ND	22.13 ± 0.02	**5.13 ± 0.1**	**3.31 ± 0.1**	8.56 ± 0.02	ND	ND
15 Day	0.47 ± 0.01	ND	ND	22.02 ± 0.01	**6.12 ± 0.1**	**0.80 ± 0.2**	9.19 ± 0.2	ND	ND
20 Day	0.63 ± 0.01	0.13 ± 0.08	ND	22.88 ± 0.01	**12.76 ± 0.1**	**1.42 ± 0.4**	9.40 ± 0.6	0.23 ± 0.03	ND


**FIGURE 5:**
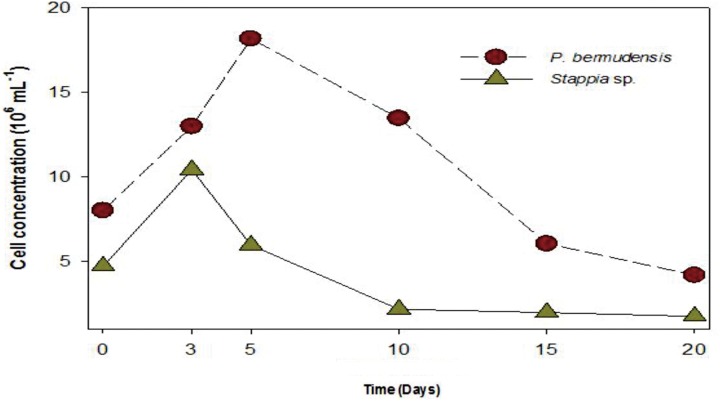
**Bacterial abundance (*P. bermudensis* and *Stappia* species) with organic source from metabolically inactive *T. striata***.

*The corresponding bacterial concentration in the experiment is shown in **Figure 5**. (ND, no detection; TP, Dissolved + Total particulate phosphate; DIP, dissolved inorganic phosphate; and DOP, dissolved organic phosphate). Bold indicates significant changes of phosphate by bacterial activity.*

The results obtained from the *Stappia* sp. treated metabolically inactive *T. striata* culture were contrasting to the *P. bermudensis* treated metabolically inactive *T. striata* culture. The DIP and DOP concentration of the *Stappia* sp. treated metabolically inactive *T. striata* culture were not detected except for 20th day with 0.23 μMol. The TP concentration in all of the experiments was moreover constant during the growth (**Table 2**). This clearly indicated that there was no possible phosphate supply by *Stappia* sp. to *T. striata* in co-cultivation system. It was found that TP of the samples analyzed increased slightly in axenic culture of metabolically inactive *T. striata* (**Table 2**). However, the variation in TP (0.47–0.63 μMol) was not considered as a significant variation during the different time interval since, the lesser variation is possible due to the interference caused by the silicate and other impurities, even though reagents were used to minimize the interference. The problems related to the sensitivity of the method and variations observed have been discussed by Grasshoff et al. (2009).

A bacterial role for phosphate exudation has been elucidated earlier and it was mentioned as one of the common functions therein to transform P source as organic phosphate and finally to inorganic phosphate by solubilizing organic phosphate (Rodríguez and Fraga, 1999). Organic phosphate could be transformed into inorganic phosphate during the interaction occurring in co-culture system. DOP was highest (up to 3.31 μM mL^-1^) at 5th day, with the highest bacterial concentration (up to 20 × 10^6^ mL^-1^) (**Figure 5**). This result also suggested that bacterial activity was invigorated due to high concentration of bacteria and involved in phosphate release. The conspicuous results may conclude that one of the functions of *P. bermudensis* promoted *Tetraselmis* growth was to supply phosphate during algal–bacterial interaction. However, *T. striata* inoculated with *Stappia* sp. did not show any significant nutrients exchange in co-culture, even though *Stappia* sp. stimulated growth of *T. striata* in O3 media. *Stappia* sp. may support other compounds for algal growth, such as chelators and phytohormones or co-operate by removing excess oxygen (Wang et al., 2016). However, in the late phase of the experiment (20–30 days), the growth promotion under vitamin B_12_-limited condition (**Figure 4**) indicates unknown significant interaction. For instance, tropodithietic acids, 2,3-butanediol and acetoin produced by members of *Roseobacter* clade, known for stimulating growth of plants (Ping and Boland, 2004; Ryu et al., 2004). In summary, *P. bermudensis* expedited growth of *T. striata* by supplying available phosphate. However, other approaches are required to unveil details of mechanism between *Stappia* sp. and *T. striata*. Furthermore, although *P. bermudensis* seems to have a phosphate solubilizing function, it may be one part of its *multi*-functions to stimulate the growth of microalgae (Kaneda, 1991).

### Evidence of Species-Specific Mutualistic Interaction

**FIGURE 6:**
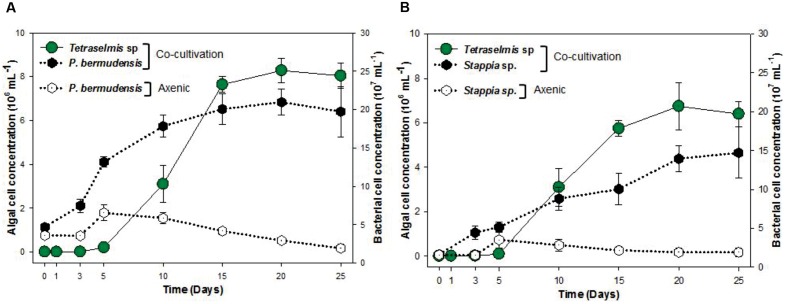
**Cell concentration of *T. striata* with bacteria** **(A)** *P. bermudensis* and **(B)** *Stappia* sp. in co-cultivation condition and axenic bacteria **(A)** *P. bermudensis* and **(B)** *Stappia* sp. during the growth in O3 media.

To understand and unveil the close mutualistic interaction between *T. striata* and TGPB (*P. bermudensis* and *Stappia* sp.), bacterial growth were compared in O3 media culture. The results indicated that it is not possible for bacteria alone to grow in O3 medium, however, both TGPB and *T. striata* were grown up to concentrations of more than 10^8^ ml^-1^ when they co-existed (**Figure 6**). These results demonstrated that *T. striata* released algal dissolved organic carbon (DOC) during the growth which is a source of carbon and energy for bacteria (Kim et al., 2014). To clarify mutualistic relationship specificity between *T. striata* and TGPB, further study with other phytoplankton was conducted to prove species specific interaction which has been known between microalgae and bacteria. Results suggested that HYYH-1409-4-3 (*P. bermudensis*) and HYYH-1409-17-3 (*Stappia* sp.) had weak or no significant growth promoting effect on *Chattonella marina*, *Amphidinium* sp., and *Dunaliella tertiolecta* except for *Heterosigma akahiwo* (**Table 1**). *Stappia* species could show positive effects on *Heterosigma akahiwo*. However, some other isolates had shown positive as well as negative effects on these microalgal species (**Table 1**). These results suggest that both *P. bermudensis* and *Stappia* sp. may have species specific communication with *T. striata*. However, the recent study showed that the synthetic mutualism may be also established between microalgae and bacteria upon significant period of co-evolution which encourages the use of these bacteria against other microalgae too upon investigation and on optimization of cell density, pH, light intensity, salinity, and nutrient conditions (de-Bashan et al., 2016). The growth promoting effect of HYYH-1409-4-3 (*P. bermudensis*) and HYYH-1409-17-3 (*Stappia* species) on the *T. striata* established in this study highlights its use for understanding mutualistic interaction as well as possible application for the products oriented economic goals.

### Economical Perspectives Applying to Algal Bacterial Interaction

Most of the modern available biotechnological culture systems would be suitable for the products that cost over $10 USD/kg (Mai et al., 2013) and bioenergy product should be even cheaper and affordable. The biomass and product yield has to be increased and the cost of production should be reduced to attain feasible price for commodity. Mutualistic bacteria associated with microalgae have the ability to fix or regenerate useful vital elements for their host algae and potentially decrease the cost of nutrient input (Wang et al., 2016). Thus, this study encourages future applicability of TGPB for mass algal cultivation system utilizing mutualistic bacterial interactions proposed as a means to improve yields and reduce costs.

## Conclusion

Microalgal bacterial interaction would be a promising breakthrough for better algal cultivation. Two bacteria (*P. bermudensis* and *Stappia* sp.) proved promising to boost biomass and lipid productivity of *T. striata* in co-cultivation system. Particularly, it is plausible that *P. bermudensis* has the ability to supply phosphate to *T. striata*. Although the exact nature of the growth promoting effects could not be pinpointed, this could be attributed to its involvement in various functions. Such bacterium-alga systems need to be evaluated under field conditions to achieve higher production of biofuel and bioproducts.

## Author Contributions

JP performed experiments, data analysis and wrote manuscript. BSP planned, guided and performed experiments. PW screened and isolated the bacteria. JK and S-HK contributed to phosphate related experiments. M-SH and SP planned experiments and advised studies.

## Conflict of Interest Statement

The authors declare that the research was conducted in the absence of any commercial or financial relationships that could be construed as a potential conflict of interest.

## Footnotes

1 http://www.bionicsro.co.kr

2 http://www.macrogen.com

## Abbreviations

DCW: dry cell weight
DIP: dissolved inorganic phosphate
DOP: dissolved organic phosphate
TGPB: *Tetraselmis striata* growth promoting bacteria
TP: total phosphate
