# Supplementary material for: Phycospheric Native Bacteria Pelagibaca bermudensis and Stappia sp. Ameliorate Biomass Productivity of Tetraselmis striata (KCTC1432BP) in Co-cultivation System through Mutualistic Interaction
Source: Front Plant Sci. 2017 Mar 6;8:289. doi: 10.3389/fpls.2017.00289 (PMC5337489; doi:10.3389/fpls.2017.00289)
Supplement: Supplementary file 1 [file Presentation_1.PPTX]

## Slide 1
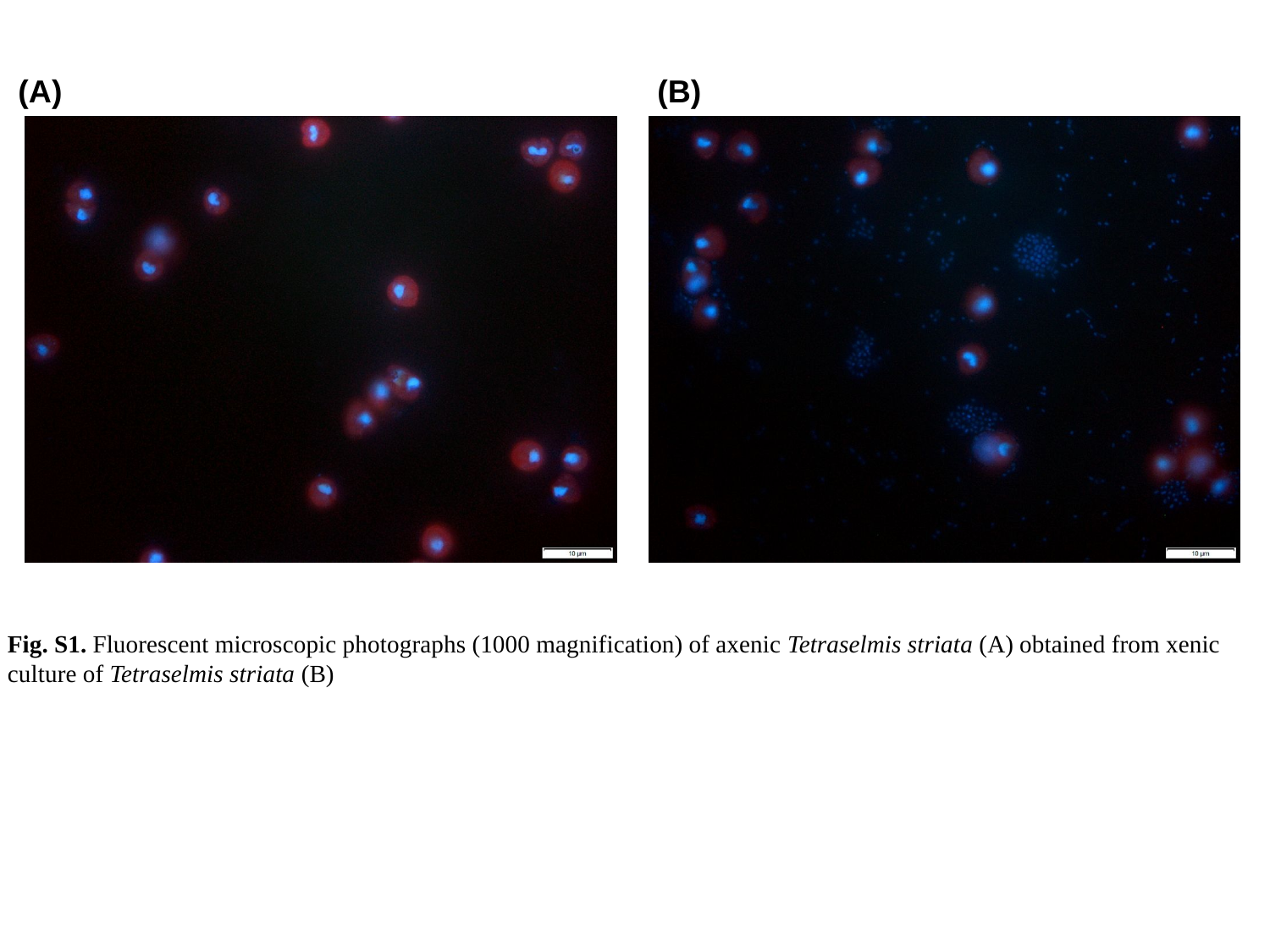

(A)
(B)

## Slide 2
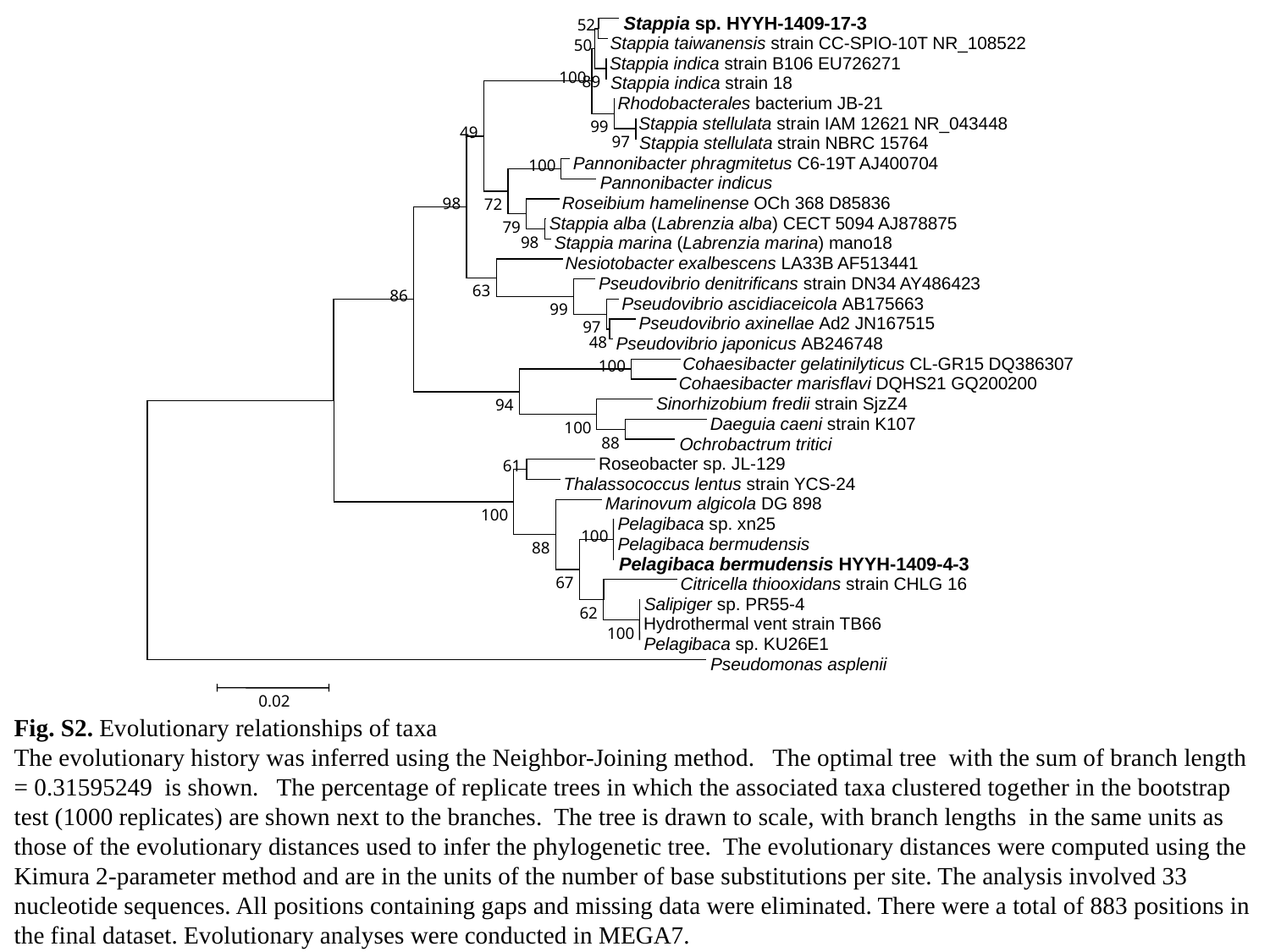

Stappia sp. HYYH-1409-17-3
52
 Stappia taiwanensis strain CC-SPIO-10T NR_108522
50
 Stappia indica strain B106 EU726271
100
89
 Stappia indica strain 18
 Rhodobacterales bacterium JB-21
 Stappia stellulata strain IAM 12621 NR_043448
99
49
 Stappia stellulata strain NBRC 15764
97
 Pannonibacter phragmitetus C6-19T AJ400704
100
 Pannonibacter indicus
 Roseibium hamelinense OCh 368 D85836
98
72
 Stappia alba (Labrenzia alba) CECT 5094 AJ878875
79
98
 Stappia marina (Labrenzia marina) mano18
 Nesiotobacter exalbescens LA33B AF513441
 Pseudovibrio denitrificans strain DN34 AY486423
63
86
 Pseudovibrio ascidiaceicola AB175663
99
 Pseudovibrio axinellae Ad2 JN167515
97
48
 Pseudovibrio japonicus AB246748
 Cohaesibacter gelatinilyticus CL-GR15 DQ386307
100
 Cohaesibacter marisflavi DQHS21 GQ200200
 Sinorhizobium fredii strain SjzZ4
94
 Daeguia caeni strain K107
100
88
 Ochrobactrum tritici
 Roseobacter sp. JL-129
61
 Thalassococcus lentus strain YCS-24
 Marinovum algicola DG 898
100
 Pelagibaca sp. xn25
100
 Pelagibaca bermudensis
88
 Pelagibaca bermudensis HYYH-1409-4-3
67
 Citricella thiooxidans strain CHLG 16
 Salipiger sp. PR55-4
62
 Hydrothermal vent strain TB66
100
 Pelagibaca sp. KU26E1
 Pseudomonas asplenii
0.02
Fig. S2. Evolutionary relationships of taxa
The evolutionary history was inferred using the Neighbor-Joining method. The optimal tree with the sum of branch length = 0.31595249 is shown. The percentage of replicate trees in which the associated taxa clustered together in the bootstrap test (1000 replicates) are shown next to the branches. The tree is drawn to scale, with branch lengths in the same units as those of the evolutionary distances used to infer the phylogenetic tree. The evolutionary distances were computed using the Kimura 2-parameter method and are in the units of the number of base substitutions per site. The analysis involved 33 nucleotide sequences. All positions containing gaps and missing data were eliminated. There were a total of 883 positions in the final dataset. Evolutionary analyses were conducted in MEGA7.

## Slide 3
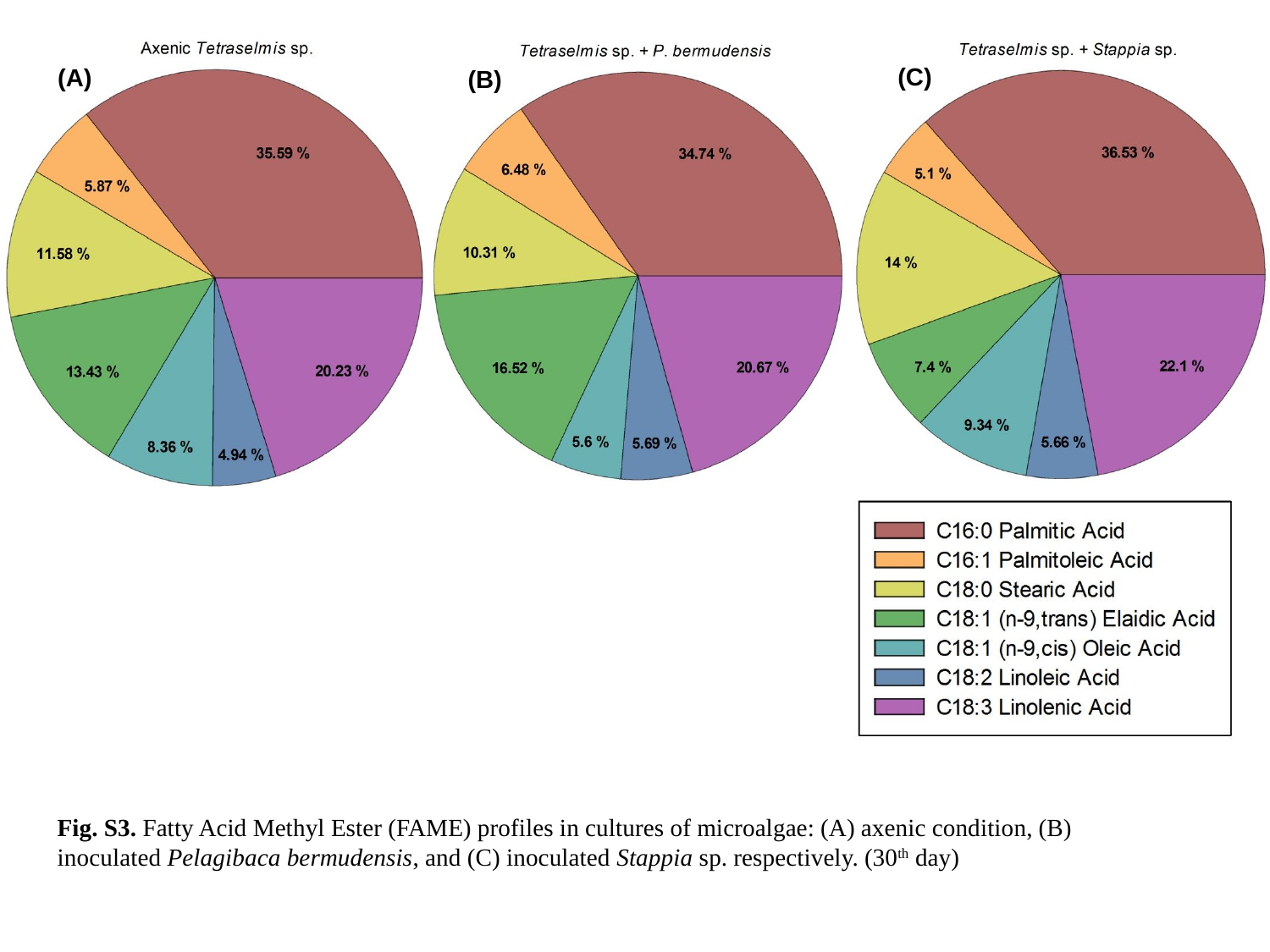

(C)
(A)
(B)
Fig. S3. Fatty Acid Methyl Ester (FAME) profiles in cultures of microalgae: (A) axenic condition, (B) inoculated Pelagibaca bermudensis, and (C) inoculated Stappia sp. respectively. (30th day)
